# Non-structural misalignments of body posture in the sagittal plane

**DOI:** 10.1186/s13013-018-0151-5

**Published:** 2018-03-05

**Authors:** Dariusz Czaprowski, Łukasz Stoliński, Marcin Tyrakowski, Mateusz Kozinoga, Tomasz Kotwicki

**Affiliations:** 1 Department of Physiotherapy, Józef Rusiecki University College in Olsztyn, Bydgoska 33, 10-243 Olsztyn, Poland; 2 Center of Body Posture, Bydgoska 33, 10-243 Olsztyn, Poland; 3 Spine Disorders Center, Rehasport Licensed Rehabilitation Center, Al. Niepodległości 4, 96-100 Skierniewice, Poland; 4 Spine Disorders and Pediatric Orthopedics Department, University of Medical Sciences, 28 Czerwca 1956 135/147 Street, 61-545 Poznań, Poland; 5 Rehasport Clinic, Górecka 30, 60-201 Poznań, Poland; 6 Department of Orthopaedics, Pediatric Orthopaedics and Traumatology, The Center of Postgraduate Medical Education in Warsaw, Konarskiego 13, 05-400 Otwock, Poland

**Keywords:** Body posture, Corrective exercises, Faults of body posture, Lordotic posture, Kyphotic posture, Flat-back posture, Sway-back posture

## Abstract

**Background:**

The physiological sagittal spinal curvature represents a typical feature of good body posture in the sagittal plane. The cervical and the lumbar spine are curved anteriorly (lordosis), while the thoracic segment is curved posteriorly (kyphosis). The pelvis is inclined anteriorly, and the lower limbs’ joints remain in a neutral position. However, there are many deviations from the optimal body alignment.

The aim of this paper is to present the most common types of non-structural misalignments of the body posture in the sagittal plane.

**Main body of the abstract:**

The most common types of non-structural misalignments of body posture in the sagittal plane are as follows: (1) lordotic, (2) kyphotic, (3) flat-back, and (4) sway-back postures. Each one may influence both the skeletal and the muscular system leading to the functional disturbance and an increased strain of the supporting structures. Usually, the disturbances localized within the muscles are analyzed in respect to their shortening or lengthening. However, according to suggestions presented in the literature, when the muscles responsible for maintaining good body posture (the so-called stabilizers) are not being stimulated to resist against gravity for an extended period of time, e.g., during prolonged sitting, their stabilizing function is disturbed by the hypoactivity reaction resulting in muscular weakness. The deficit of the locomotor system stability triggers a compensatory mechanism—the stabilizing function is overtaken by the so-called mobilizing muscles. However, as a side effect, such compensation leads to the increased activity of mobilizers (hyperactivity) and decreased flexibility, which may finally lead to the pathological chain of reaction within the musculoskeletal system.

**Conclusions:**

There exist four principal types of non-structural body posture misalignments in the sagittal plane: lordotic posture, kyphotic posture, flat-back posture, and sway-back posture. Each of them can disturb the physiological loading of the musculoskeletal system in a specific way, which may lead to a functional disorder.

When planning postural corrective exercises, not only the analysis of muscles in respect to their shortening and lengthening but also their hypoactivity and hyperactivity should be considered.

## Background

### Human body posture

Human posture is commonly understood as the relationship between human body parts in the upright position. Particular body parts, such as the head and neck, the trunk, and the upper and lower limbs, are involved in the final body posture. A *good body posture* is considered (1) ergonomically advantageous while standing, (2) mechanically effective while moving, and (3) supportive for the normal function of internal organs. Body posture is described and considered in three reference planes: sagittal, coronal, and transversal [1, 2]. Kendall et al. proposed a definition of good human posture: “good posture is that state of muscular and skeletal balance which protects the supporting structures of the body against the injury or progressive deformity, irrespective of the attitude (erect, lying, squatting or stooping) in which these structures are working or resting. Under such conditions, the muscles will function most efficiently, and the optimum positions are afforded for the thoracic and abdominal organs” [3]. Such a comprehensive definition of body posture will not be used in this paper since the authors focused on the description of human posture in the upright standing position.

*Poor posture* is an imprecise term commonly used in the clinical practice to describe a relationship between various body parts which may be considered as faulty and which could stretch the spectrum from the non-perfect to pathological posture. It is postulated that poor posture can produce an increased strain on the supporting structures and less-efficient balance of the body over its base of support [3].

The most difficult task of describing *good body posture* concerns the sagittal plane alignment, while both the coronal and the transversal planes are usually considered symmetrical. In fact, the human being is symmetrical neither in the coronal nor in the transversal plane [3–5]. However, this simplification is used in this paper for the clear presentation of the sagittal plane alignment.

The *physiological sagittal spinal curvature* represents a typical feature of good body posture in the sagittal plane. The cervical and the lumbar spine are curved anteriorly (*lordosis*), while the thoracic segment is curved posteriorly (*kyphosis*). The head remains horizontal, which denotes that the eye level corresponds to the horizontal plane, while the chin is positioned just above the sternum. The pelvis is inclined anteriorly, and the lower limb joints remain in a neutral position [1–3].

**Fig. 1:**
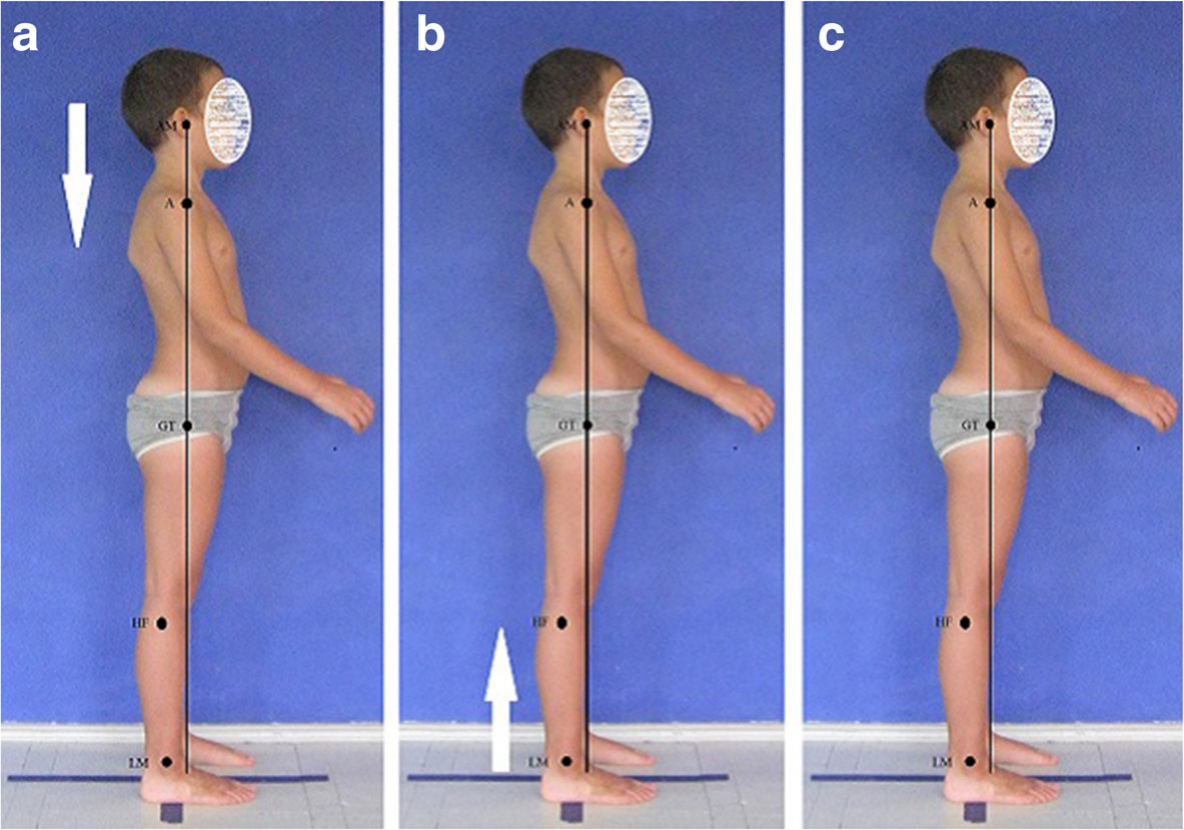
Good body posture in a 8-year-old boy—the head line (**a**) and the base line (**b**) overlaps each other (**c**). Note: AM—external auditory meatus; A—acromion; GT—greater trochanter; HF—head of fibula; LM—lateral malleolus

The optimal body posture should represent the following alignment: the *head line*, beginning at the external auditory meatus (or at the mastoid process of the temporal bone), should run vertically through the acromion, the lumbar vertebral bodies, the promontory, then slightly posteriorly to the hip joint axis, slightly in front of the knee joint axis, and finish at the lateral malleolus or slightly in front of it. The course of this line in a good body posture overlaps the *base line* joining the center of gravity with the central point of the supporting area (Fig. 1) [4–7].

As presented above, the detailed description of a good body posture in the sagittal plane is not explicit. Moreover, characterizing the deviations from a good posture can be ambiguous. The aim of this paper is to present the most common types of non-structural misalignments of body posture in the sagittal plane.

### Non-structural versus structural misalignments of body posture

From a clinical point of view, the disturbances of human posture can be classified as non-structural or structural. The non-structural pathologies represent the main topic of this article and will be discussed in detail. The structural misalignments comprise specific clinical entities: idiopathic scoliosis, Scheuermann juvenile kyphosis, congenital vertebral malformation, sequels of spine osteomyelitis, spondylolisthesis, and other clinical entities that produce disorders of body posture, e.g., thoracic hyperkyphosis, flat back, and pelvis malposition. The said body posture disorders are known as “structural disorders,” as this term indicates the presence of morphological abnormalities within the bones and soft tissues (fascia, muscles, ligaments, tendons). Additionally, structural misalignments reveal a more severe clinical problem as they are less flexible and less prone to correction compared to the non-structural disorders. They require specific diagnostic and therapeutic approach and are not discussed in this paper apart from the differential diagnosis issue.

**Fig. 2:**
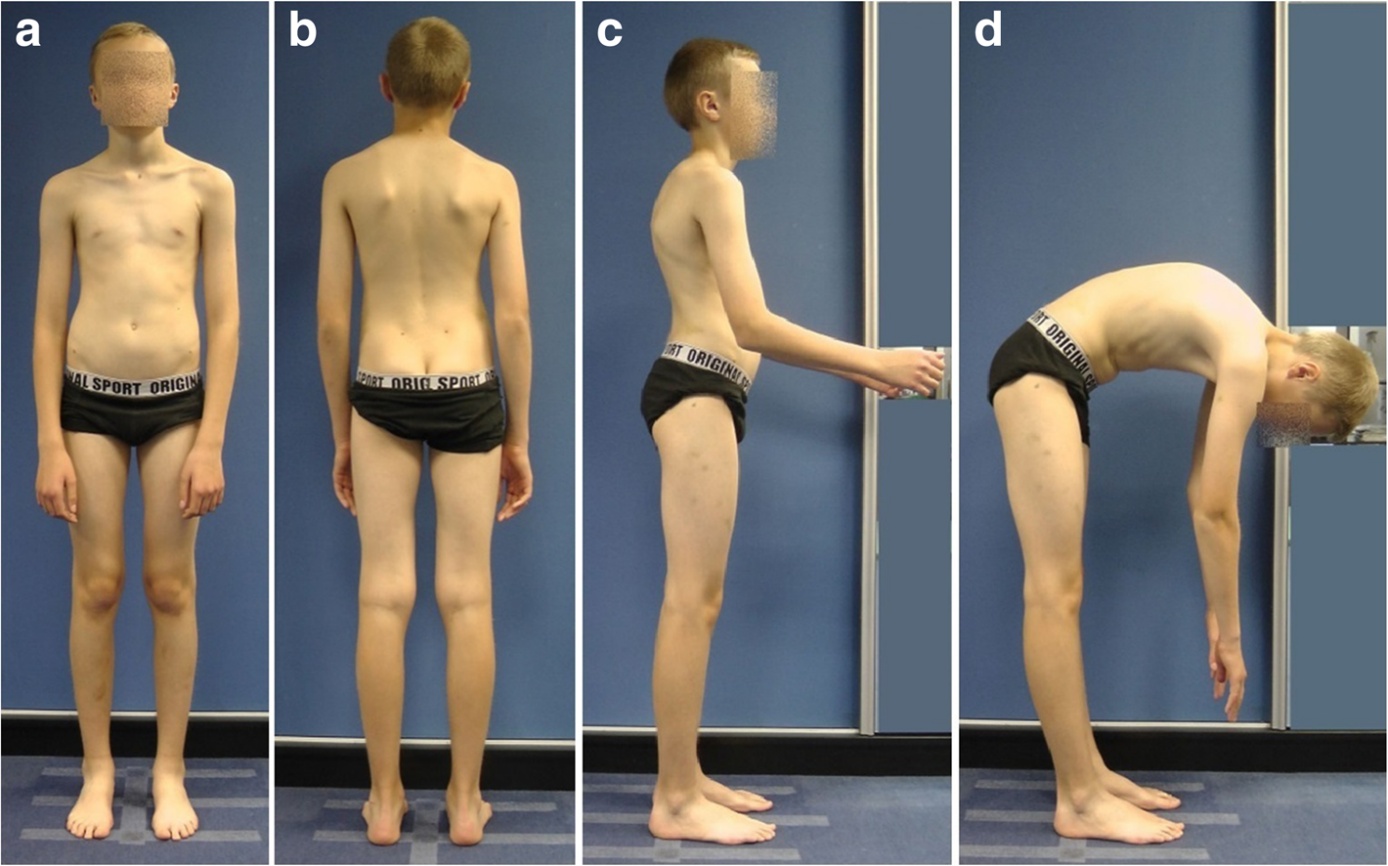
A 12-year-old boy with non-structural sagittal misalignment of body posture: postural thoracic hyperkyphosis. **a** Front view. **b** Back view. **c** Side view. **d** Forward bend view

**Fig. 3:**
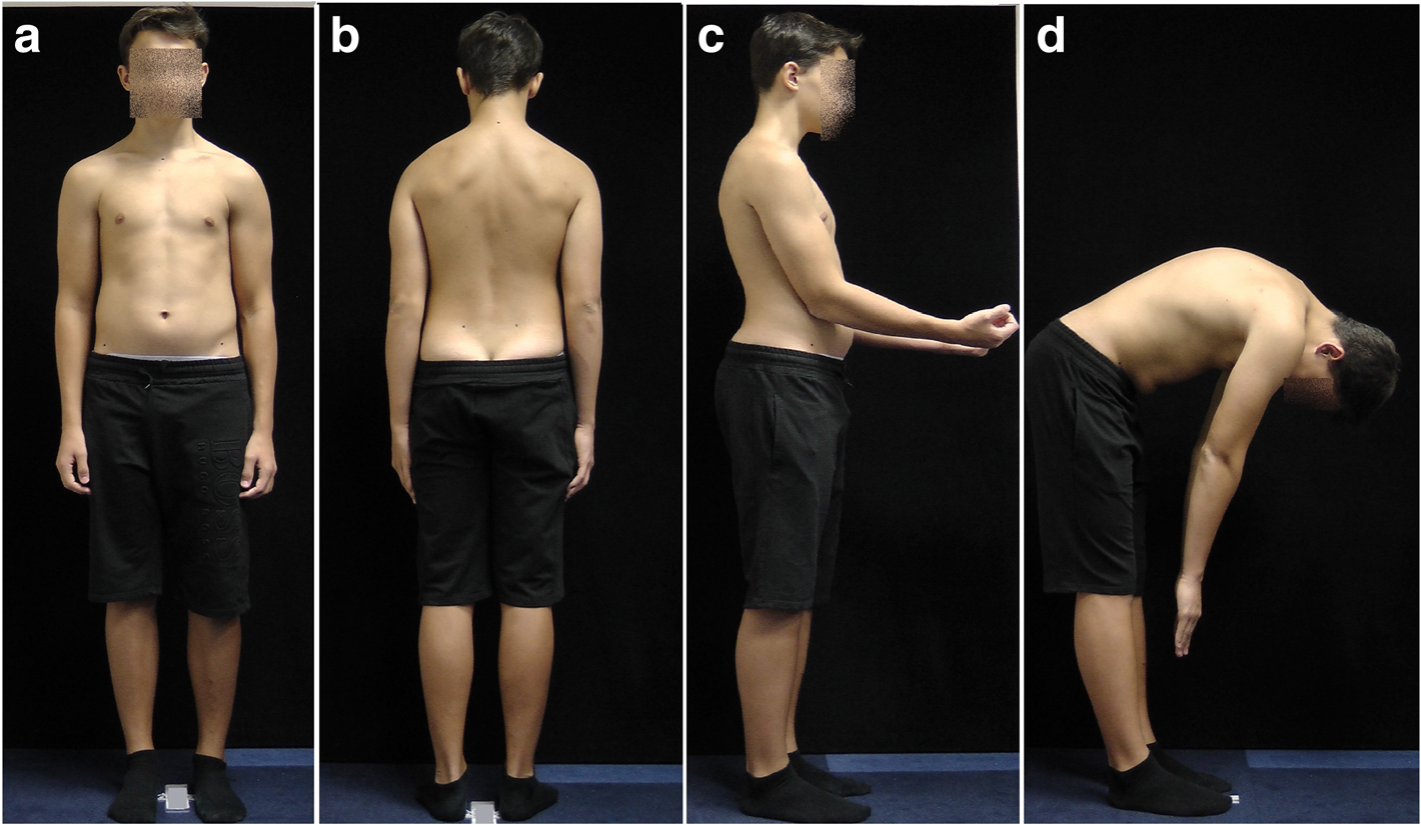
A 14-year-old boy with structural sagittal misalignment of body posture: structural thoracic hyperkyphosis. **a** Front view. **b** Back view. **c** Side view. **d** Forward bend view

The clinical appearance of children with non-structural versus structural (e.g., Scheuermann disease) disturbances of body posture may be similar (Fig. 2 versus Fig. 3). Two boys, aged 12 and 14 respectively, diagnosed with the kyphotic posture (increased thoracic kyphosis, protraction of the head and shoulders), are presented in Figs. 2 and 3. Figure 2 shows the kyphotic posture reasonable due to the non-structural pathology, namely the combination of an incorrect postural habit and muscles’ hypo- and hyperactivity. Figure 3 presents the kyphotic posture due to the structural thoracic hyperkyphosis, which is a structural spinal deformity.

**Fig. 4:**
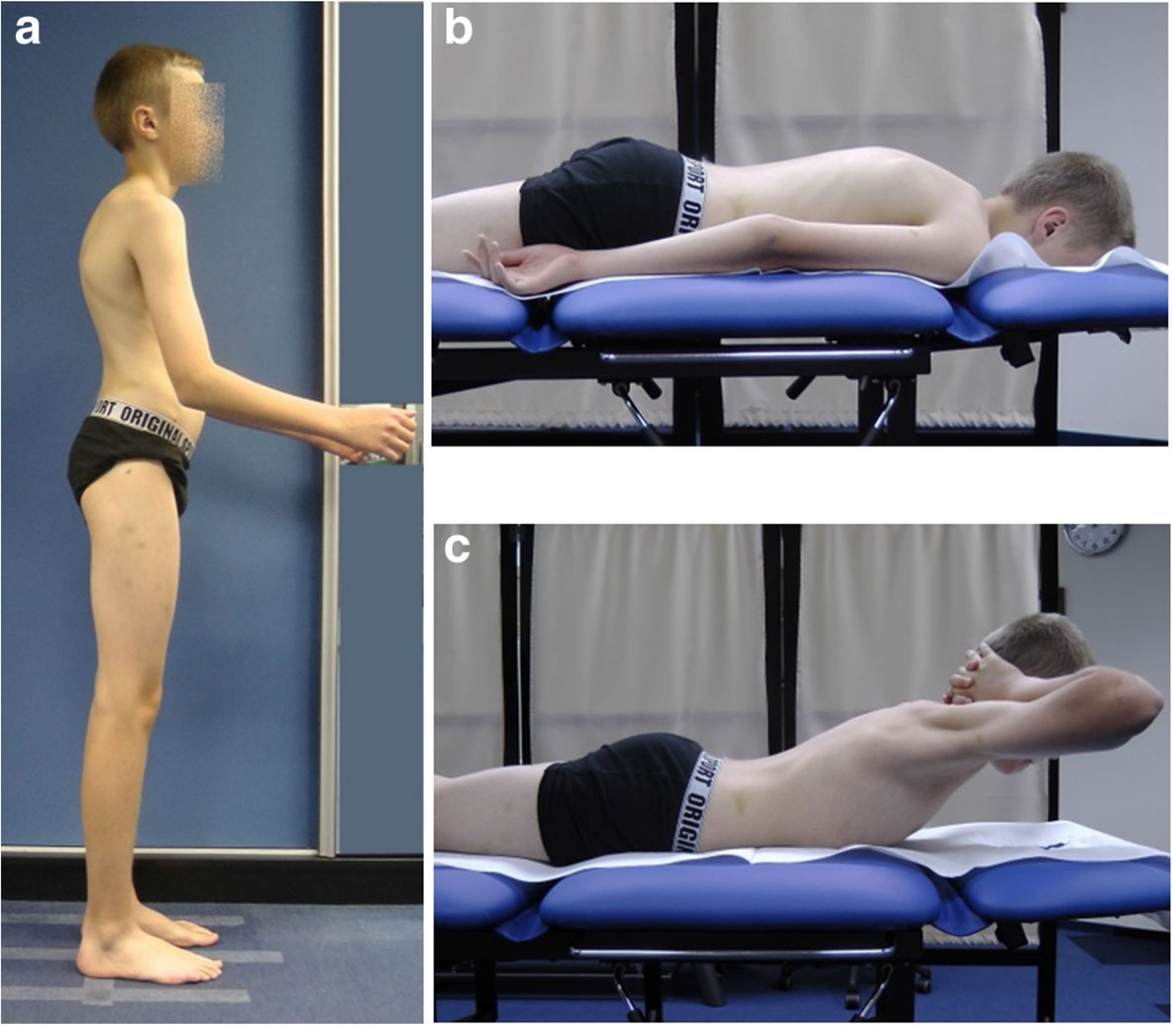
A 12-year-old boy with thoracic hyperkyphosis developing in habitual standing position. **a** Habitual standing position, lateral view. **b** Prone habitual lying position reveals thoracic hyperkyphosis. **c** Active trunk extension causes correction—flattening of thoracic hyperkyphosis

**Fig. 5:**
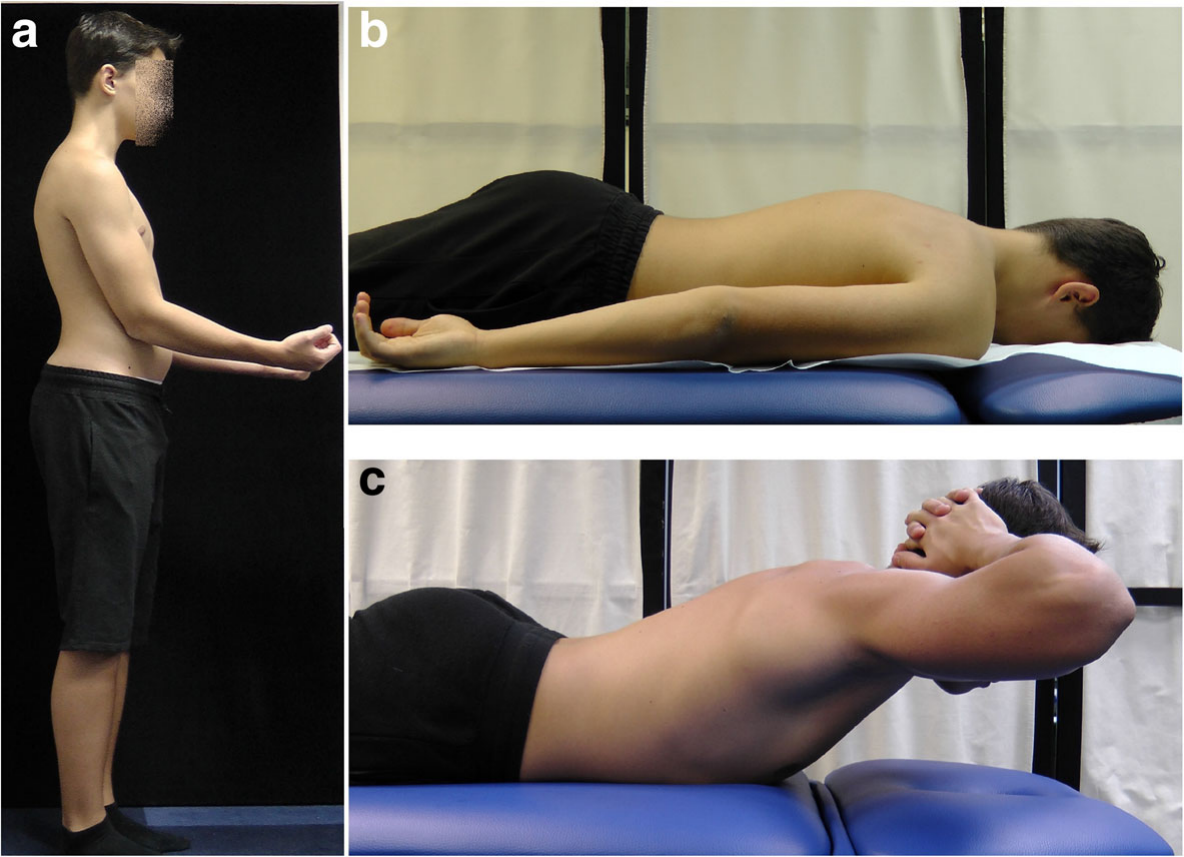
A 14-year-old boy with structural thoracic hyperkyphosis. **a** Habitual standing position, lateral view. **b** Lying prone position reveals maintaining thoracic hyperkyphosis. **c** Active trunk extension does not decrease the thoracic hyperkyphosis

Differential diagnosis represents an important part of the evaluation of every child addressed for the so-called poor posture. Despite the modern imaging techniques, including digital whole-body radiography, computed tomography, or nuclear magnetic resonance, the basic clinical examination retains its value. For example, the functional testing allows assessing the flexibility of thoracic hyperkyphosis which reveals good in non-structural (Fig. 4a–c) versus poor in structural misalignment (Fig. 5a–c).

### Non-structural sagittal misalignments of body posture

#### Principal types of sagittal postural misalignments

The most common types of non-structural misalignments of body posture in the sagittal plane in both children and adults are: (1) *lordotic posture*, (2) *kyphotic posture* which can sometimes coexist with the lordotic one as a *kyphotic-lordotic posture*, (3) *flat-back posture*, and (4) *sway-back posture* [4, 7, 8]. The biomechanical analysis of body alignment and the functional analysis of the muscles involved in each type of faulty posture reveal the muscle groups that remain the target for corrective management. Therefore, before the detailed description of particular types of faulty postures is given, the concept of functional muscle classification will be presented.

#### Functional muscle classification by Bergmark and Richardson in the context of body posture

Bergmark [9] and Richardson et al. [10] reported on the functional specificity of skeletal muscles, expressed in a normal condition and response to stress. Many studies confirmed that individual skeletal muscles react differently to common events, such as injury of the associated joint, presence, or lack of gravitational load or specific patterns of use (e.g., ballistic exercises) [11–17], namely by *reflectory inhibition* or *reflectory excitation*. *Reflectory inhibition* results in muscle *hypoactivity* which may manifest clinically as *muscle weakness*. *Reflectory excitation* results in muscle *hyperactivity* which may manifest clinically as *reduced flexibility* [11–18]. Such reduced flexibility is usually reported on the clinical examination as muscle shortening even though it does not involve the factual *shortening* of muscular fibers (contracture), which will be explained in the further part of the paper.

#### Muscle groups maintaining good body posture

Bergmark [9] and Richardson et al. [10] proposed to classify the skeletal muscles into two groups: (1) *mono-articular*, also called local muscles or stabilizers, and (2) *multi-articular*, also called global or stabilizer/mobilizer muscles, depending on the subgroup (see below) [5, 9, 10]. According to the authors, the appropriate cooperation between these two muscle groups allows transferring the load from the thorax to the pelvis safely through the stabilized spinal segments and to minimize forces applied to the lumbar spine during functional activities [5, 6, 9, 10].

According to Bergmark [9] and Richardson et al. [10], the *local mono-articular* group comprises deep trunk muscles: multifidus, transversus abdominis, interspinalis, intertransversalis, semispinalis, posterior portion of the internal oblique, medial fibers of quadratus lumborum, the central portion of the erector spinae, diaphragm, and the muscles of the pelvic floor [9, 10]. These muscles are linked with joint stabilization, and they are capable of controlling the position of the joints or spinal segments. The stabilizers are responsible for preventing from local shifts of a particular spinal segment and provide segmental 3-D stability to maintain the global mechanical stability of the whole spine [2, 6, 8, 10, 19]. In response to stress, the local muscles are likely to undergo reflectory inhibition (hypoactivity). It may be caused by injury to the associated joint, ballistic repetitive exercises, or lack of use and lack of gravitational load [9–13, 15].

The *global multi-articular* muscles comprise large muscles which tend to be situated superficially in the trunk and the limbs. This muscle group provides the function of both stabilizing and force-generating moments in several joints at the same time. These muscles are considered phylogenetically the oldest [10, 11]. The global muscles are divided into two subgroups: the stabilizers and the mobilizers.

The *global stabilizers* comprise the antigravity muscles responsible for maintaining the erected posture. This group of muscles includes trapezius (middle and lower part), erector spinae (lumbar part), iliacus, gluteus maximus, gluteus medius, adductor magnus, and adductor brevis. These muscles are responsible for stabilizing the joint position while the joint movement is being performed [5, 6, 9–11].

The *mobilizers* comprise the muscles not related to antigravity postural action, e.g., erector spinae (thoracic part), rectus abdominis, external abdominal oblique, the anterior portion of internal abdominal oblique, the lateral portion of quadratus lumborum, psoas, hamstrings, tensor fasciae latae, rectus femoris, and adductor longus. These muscles are basically responsible for performing active movements in joints [5–11, 20].

#### Muscle groups functioning in faulty body posture

Exposure of the human body to gravity forces, e.g., when standing or walking, is necessary to ensure proper activity of the skeletal muscles responsible for maintaining good body posture. When these muscles are not stimulated to resist gravity for an extended period, e.g., during prolonged sitting or lying, their stabilizing function is disturbed by the hypoactivity reaction resulting in muscular weakness and atrophy. The deficit of the locomotor system stability triggers a compensatory mechanism—the stabilizing function is overtaken by the mobilizing muscles. However, as a side effect, such compensation leads to mobilizers’ increased activity (hyperactivity) and, subsequently, their decreased flexibility [7, 10, 11, 16, 18, 21, 22], which may finally lead to a pathological chain of reactions within the musculoskeletal system, as described below (Figs. 12, 13, 14 and 15).

### Lordotic posture

#### Posture description

**Fig. 6:**
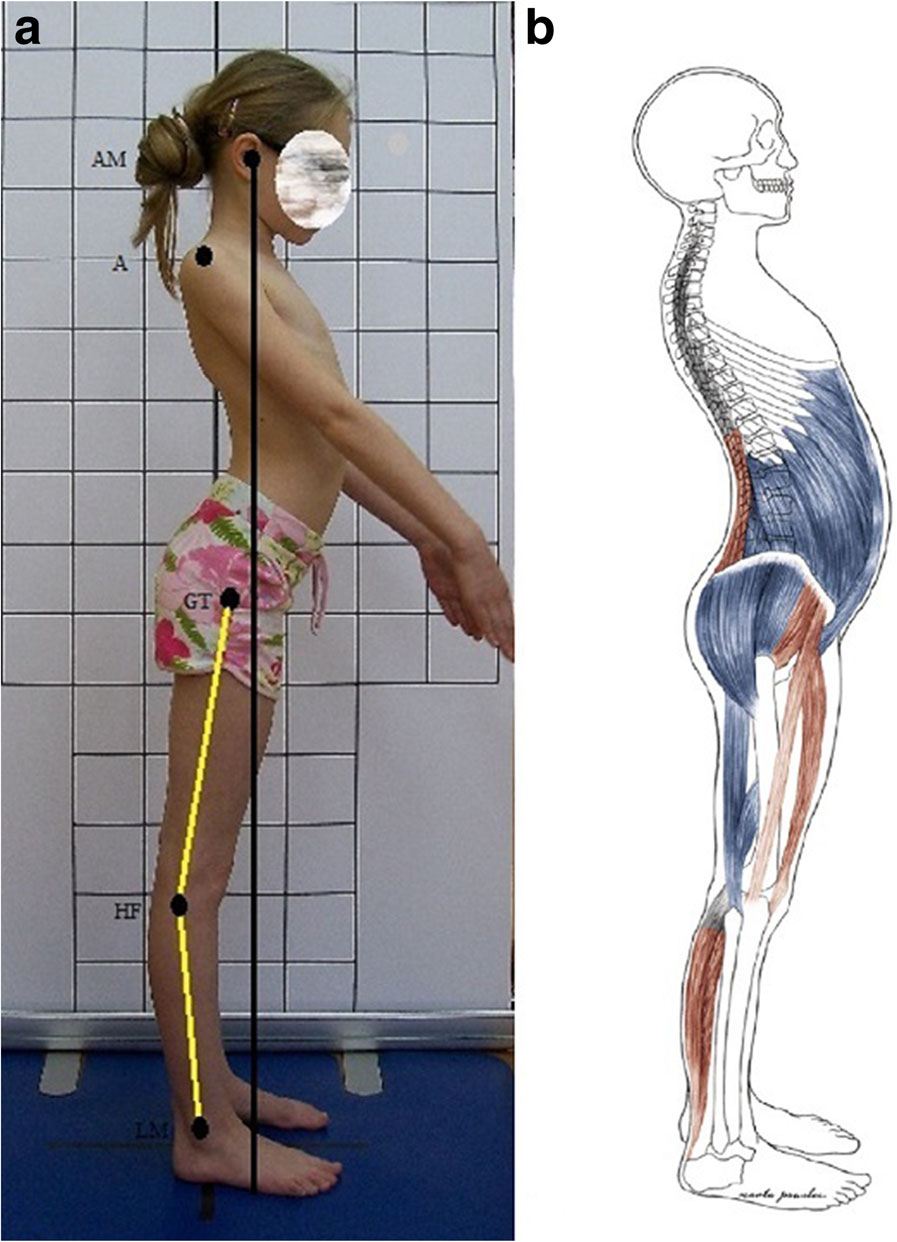
Lordotic posture in a 9-year-old girl. **a** Habitual standing, lateral view, note the hyperextension of the knees and plantar flexion of the feet. **b** Corresponding schematic representation of the shortened (red) and lengthened (blue) skeletal muscles. Note: AM—external auditory meatus; A—acromion; GT—greater trochanter; HF—head of fibula; LM—lateral malleolus

The lordotic posture represents a faulty posture that differs from the good one by the following: (1) increased lumbar lordosis and (2) increased pelvic anteversion (anterior tilt) (Fig. 6). Increased anterior tilt of the pelvis leads to increased flexion of hip joints. The knees can be in hyperextension and, due to this knee position, the plantar flexion of the feet occurs (Fig. 6) [3, 7, 8, 23].

In the lordotic posture the *head line* runs down posteriorly to lumbar vertebral bodies, passing near the intervertebral facet joints, which results in extensory overloading within the facets. The head line is also anterior to the knee joint axis, which leads to the overloading of the anterior knee compartment (Fig. 6). The *head line* may overlap the *base line,* or in the case of head protraction, it may run in front of it [3, 7, 8]. The description of the lordotic posture is given in Table 1.

**Table 1:** The position of body parts in the lordotic posture

Part of the body	Position
Head	Neutral
Cervical spine	Normal curve = physiologically convex anteriorly (lordosis)
Thoracic spine	Normal curve = physiologically convex posteriorly (kyphosis)
Lumbar spine	Hyperextended (hyperlordosis)
Pelvis	Increased anterior tilt
Hip joints	Relatively flexed
Knee joints	Hyperextended
Ankle joints	Plantar flexed

#### Functional state of muscles in the lordotic posture

The abdominal muscles, gluteus maximus, posterior part of gluteus medius, and hamstrings are lengthened [3]. The stabilizers, mainly the gluteus maximus, are hypoactive. This, in turn, generates the hyperactivity of hamstrings that compensate the gluteus maximus in its function of stabilizing the pelvis and hip joints [10, 11].

The shortened muscles comprise quadratus lumborum as well as one-joint and two-joint hip flexors, namely the iliopsoas, rectus femoris, and tensor fasciae latae, respectively. However, from a clinical point of view, iliopsoas should be analyzed as two functionally independent muscles for the iliacus and the psoas, because each of them may be either hypo- (usually iliacus) or hyperactive (usually psoas). By the same token, quadratus lumborum comprises two functionally distinguished parts: the medial and the lateral portion. The medial portion of quadratus lumborum is responsible for spine stabilization and has a tendency to hypoactivity, while the lateral portion, related to trunk movements, has a tendency to hyperactivity (Fig. 6) [9, 10, 24].

The erector spinae is worthy of special attention as, according to both the literature and the biomechanical analysis of the standing posture, this muscle is likely to present shortening in the lumbar part of the spine [3]. However, the authors’ experience reveals that this muscle is rarely shortened. We suspect that this phenomenon is a consequence of the lifestyle—spending the vast time in flexed sitting position [25, 26], so the lumbar part of erector spinae is constantly stretched. In turn, both standing and sitting position favors the shortening of hip flexors.

As a result of knee hyperextension and feet plantar flexion, the triceps surae may be shortened, including hypoactive soleus and hyperactive gastrocnemius (Table 2) [3, 9, 10].

**Table 2:** Functional characteristics of muscles in the lordotic posture

Muscle	Lengthened	Shortened	Hypoactive	Hyperactive
Rectus abdominis	+			+
Abdominal internal oblique (anterior part)	+			+
Abdominal internal oblique (posterior part)	+		+	
Abdominal external oblique	+			+
Gluteus maximus	+		+	
Gluteus medius (posterior part)	+		+	
Hamstrings	+			+
Erector spinae part lumbar (in sitting)	+		+	
Erector spinae part lumbar (in standing)		+	+	
Quadratus lumborum (medial part)		+	+	
Quadratus lumborum (lateral part)		+		+
Iliacus		+	+	
Psoas		+		+
Two-joint hip flexors		+		+
Gastrocnemius		+		+
Soleus		+	+	

Note: the symbol “+” means that the muscle meets a certain criteria

### Kyphotic posture

#### Posture description

**Fig. 7:**
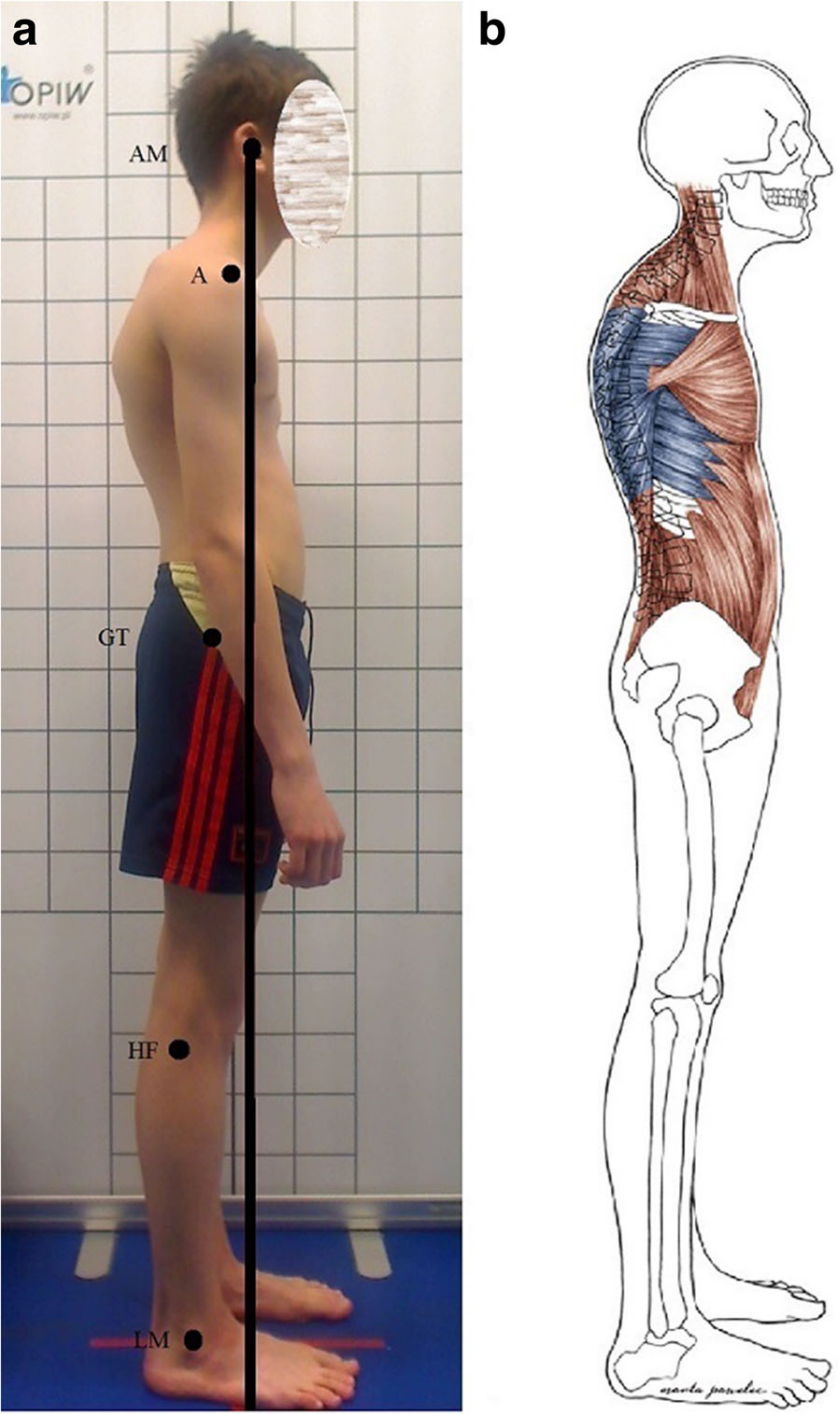
Kyphotic posture in a 13-year-old boy. **a** Habitual standing, lateral view. **b** Corresponding schematic representation of the shortened (red) and lengthened (blue) skeletal muscles. Note: AM—external auditory meatus; A—acromion; GT—greater trochanter; HF—head of fibula; LM—lateral malleolus

The kyphotic posture represents a faulty posture that differs from the good one by the following: (1) increased thoracic kyphosis, (2) head protraction, (3) flattened or reversed lower cervical lordosis, (4) increased upper cervical lordosis, and (5) protraction of shoulders and scapulae (Fig. 7) [3, 7, 8].

In the kyphotic posture, the *head line* is shifted anteriorly to the thoracic spine, lumbar vertebral bodies, and hip and knee joint axis. The *base line* usually runs at the back of the head line (Fig. 7) [3, 7]. The description of the kyphotic posture is shown in Table 3.

**Table 3:** The position of body parts in the kyphotic posture

Part of the body	Position
Head	Protracted (moved forward)
Cervical spine	Upper part: extended (hyperlordosis) Lower part: flexed (hypolordosis or kyphosis)
Scapulae	Abducted (moved laterally)
Shoulders	Protracted (moved forward)
Thoracic spine	Increased flexion (hyperkyphosis)
Chest	Tilted downward, sometimes flattened
Sternum	Tilted downward
Thoracic outlet	Increased obliquity
Lumbar spine	Neutral
Pelvis	Neutral
Hip joints	Neutral
Knee joints	Neutral
Ankle joints	Neutral

#### Functional state of muscle in the kyphotic posture

In the kyphotic posture, the thoracic part of the erector spinae, rhomboids, serratus anterior, and the lower and middle parts of trapezius muscle are lengthened [3, 7].

The shortened muscles in the kyphotic posture are as follows: suboccipital, sternocleidomastoid, scaleni, pectoralis major, pectoralis minor, and latissimus dorsi [3, 7]. Nevertheless, the latissimus dorsi may be shortened only in its part located close to the muscle insertion at the shoulder girdle (the crest of the lesser tubercle of the humerus) because of the shoulder protraction and internal rotation of the arms. On the other hand, the medial part of the latissimus dorsi may be lengthened due to increased thoracic kyphosis.

It is also worth taking a closer look at abdominal muscles. As a result of chest tilting, these muscles can be shortened, which has to be taken into consideration while selecting corrective exercises (Fig. 7) (Table 4).

**Table 4:** Functional characteristics of muscles in the kyphotic posture

Muscle	Lengthened	Shortened	Hypoactive	Hyperactive
Erector spinae (thoracic part)	+			+
Rhomboideus major and minor	+		+	
Serratus anterior	+		+	
Trapezius (middle and lower parts)	+		+	
Latissimus dorsi (medial part)	+			+
Suboccipital		+		+
Sternocleidomastoid		+		+
Scaleni		+		+
Latissimus dorsi (area of insertion)		+		+
Trapezius (superior part)		+		+
Pectoralis minor and major		+		+
Rectus abdominis		+		+
Abdominal internal oblique (anterior part)		+		+
Abdominal internal oblique (posterior part)		+	+	
Abdominal external oblique		+		+

Note: the symbol “+” means that the muscle meets a certain criteria

### Kyphotic-lordotic posture

**Fig. 8:**
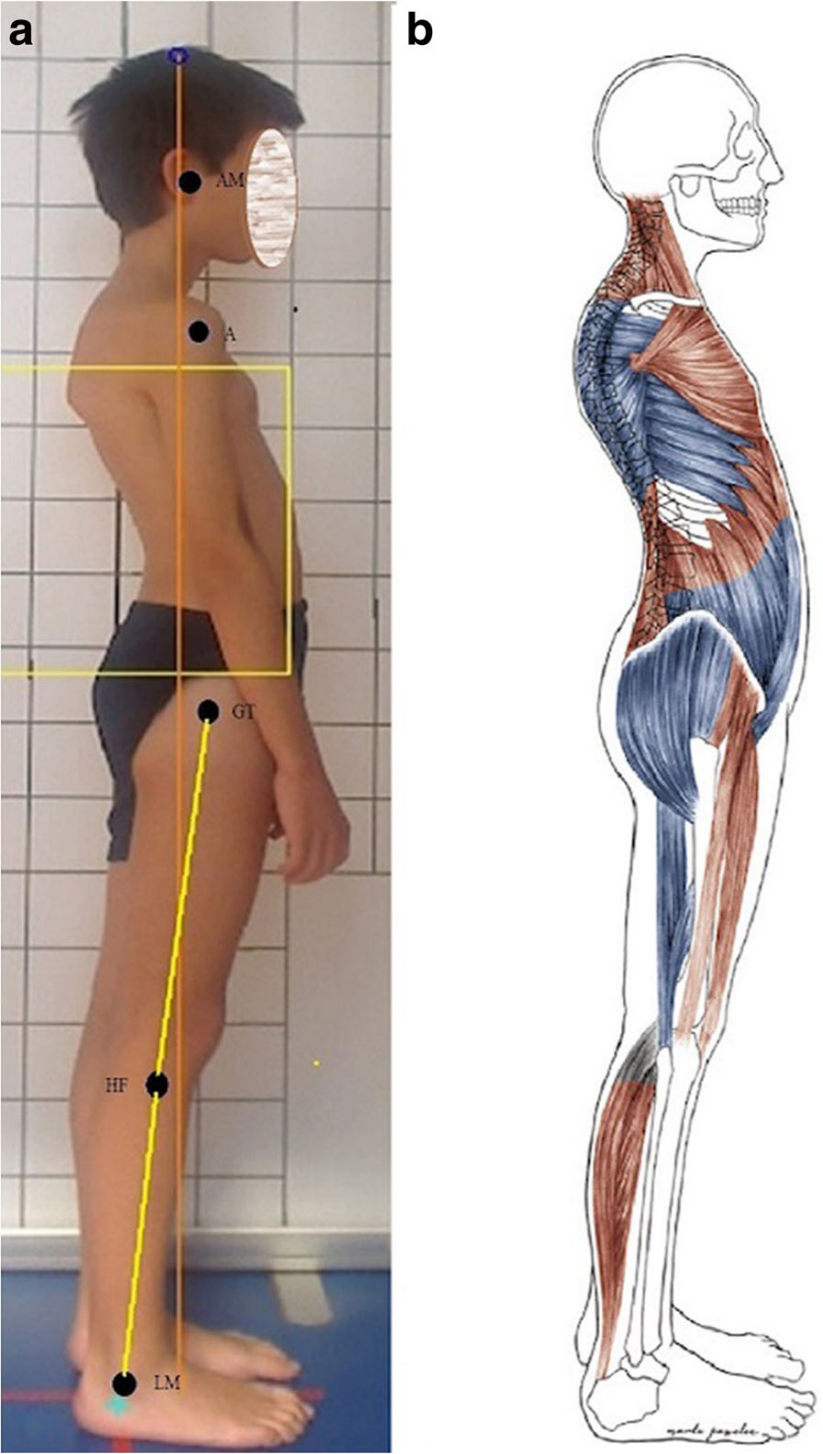
Kyphotic-lordotic posture in a 12-year-old boy. **a** Habitual standing, lateral view. **b** Corresponding schematic representation of the shortened (red) and lengthened (blue) skeletal muscles. Note: AM—external auditory meatus; A—acromion; GT—greater trochanter; HF—head of fibula; LM—lateral malleolus

In some individuals, the combination of the two aforementioned sagittal misalignments can be noted in the form of kyphotic-lordotic posture (Fig. 8) [3]. In this case, the influence of kyphotic and lordotic posture on the musculoskeletal system is combined [3, 7].

The authors would like to emphasize that difficulties in planning corrective exercises can occur in the kyphotic-lordotic posture. For instance, in lordotic posture, the abdominal muscles are lengthened and therefore should be shortened, while it is not recommended in the kyphotic posture. Although providing therapeutic schemata extends beyond the content of this paper, this example illustrates accurately the need for a nuanced physiotherapy: shortening the lower part of the abdominals (e.g., by moving upward their attachment to the pubic symphysis and iliac crest) while increasing the length of their upper part (Fig. 8).

### Flat-back posture

#### Posture description

**Fig. 9:**
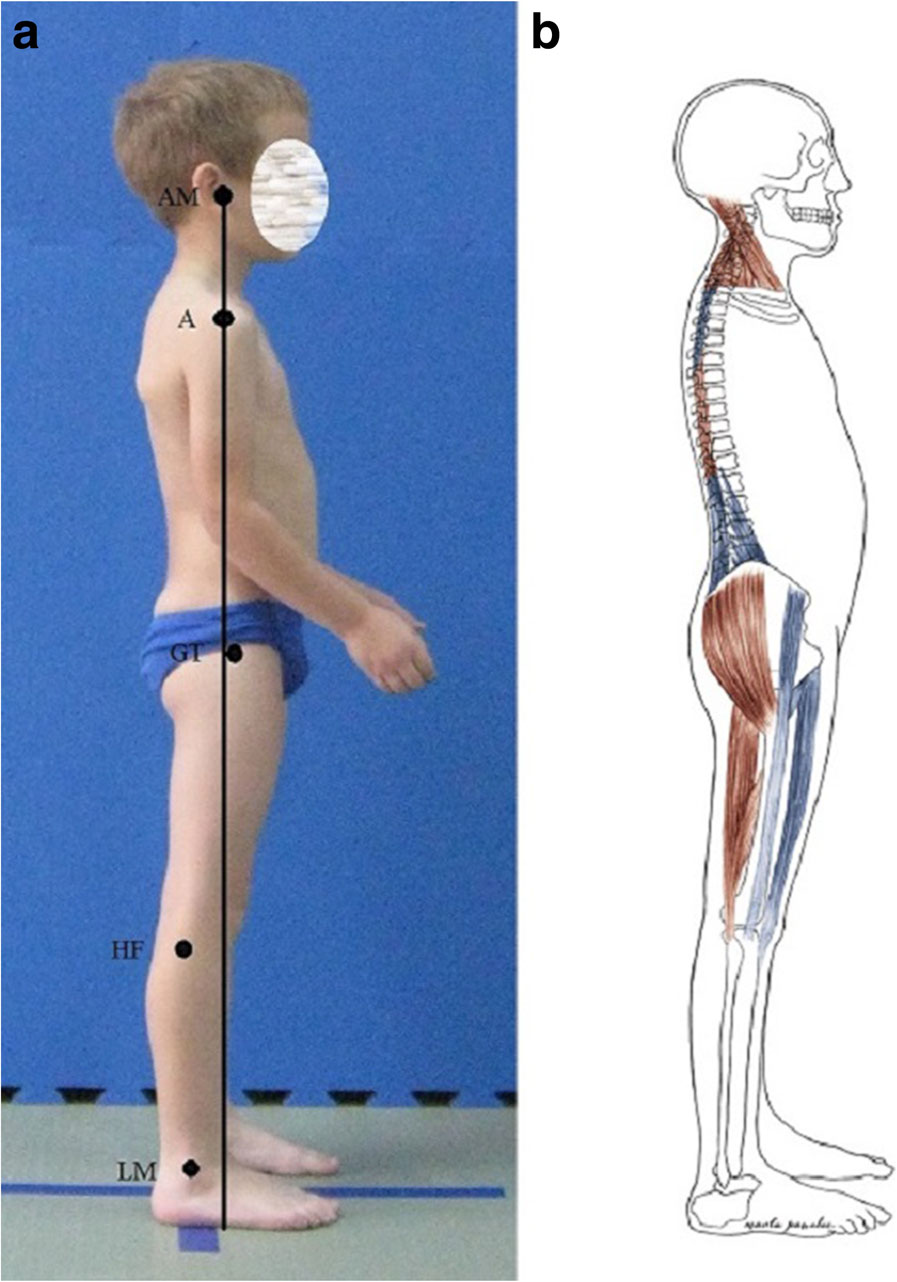
Flat-back posture in a 9-year-old boy. **a** Habitual standing, lateral view. **b** corresponding schematic representation of the shortened (red) and lengthened (blue) skeletal muscles. Note: AM—external auditory meatus; A—acromion; GT—greater trochanter; HF—head of fibula; LM—lateral malleolus

The flat-back posture represents a faulty posture that differs from the good one by the following: (1) flattened lumbar lordosis and (2) flattened lower part of thoracic kyphosis. Moreover, increased kyphosis in the upper part of the thoracic region as well as kyphotisation of the cervico-thoracic junction may be present (Fig. 9). Pelvis remains in a neutral position or in a decreased anterior tilt [3, 7, 8, 27].

In the flat-back posture, the head line and the base line usually overlap and pass anteriorly to the lumbar vertebral bodies (leading to their flexion overload) and posterior to the hip joint axis (Fig. 9). The head may be moved anteriorly to the base line (Table 5) [3, 7].

**Table 5:** The position of body parts in the flat-back posture

Part of the body	Position
Head	Neutral or protracted (moved forward)
Cervical spine	Upper part: extended (hyperlordosis) Lower part: flexed (hypolordosis or kyphosis)
Thoracic spine	Upper part: increased flexion (hyperkyphosis) Lower part: straight (hypokyphosis)
Lumbar spine	Flexed (hypolordosis)
Pelvis	Neutral or decreased anterior tilt
Hip joints	Neutral or extended when decreased anterior tilt of pelvis occurs
Knee joints	Neutral
Ankle joints	Neutral

#### Functional state of muscles in the flat-back posture

The muscles which are usually lengthened in this posture include erector spinae (lumbar part), one-joint hip flexors (iliacus, psoas), and two-joint hip flexors (rectus femoris, tensor fasciae latae). Iliacus is usually hypoactive, while psoas is hyperactive. Two-joint hip flexors are hyperactive [3, 9, 10].

Gluteus maximus is shortened and hypoactive; hamstrings are also shortened yet hyperactive (Table 6, Fig. 9) [3, 9, 10].

**Table 6:** Functional characteristics of muscles in the flat-back posture

Muscle	Lengthened	Shortened	Hypoactive	Hyperactive
Erector spinae part thoracic (upper part)	**+**			**+**
Erector spinae (lumbar part)	+		+	
Iliacus	+		+	
Psoas	+			+
Two-joint hip flexors	+			+
Suboccipital		+		+
Sternocleidomastoid		+		+
Scaleni		+		+
Erector spinae part thoracic (lower part)		+		+
Gluteus maximus		+	+	
Hamstrings		+		+

Note: the symbol “+” means that the muscle meets a certain criteria

### Sway-back posture

#### Posture description

**Fig. 10:**
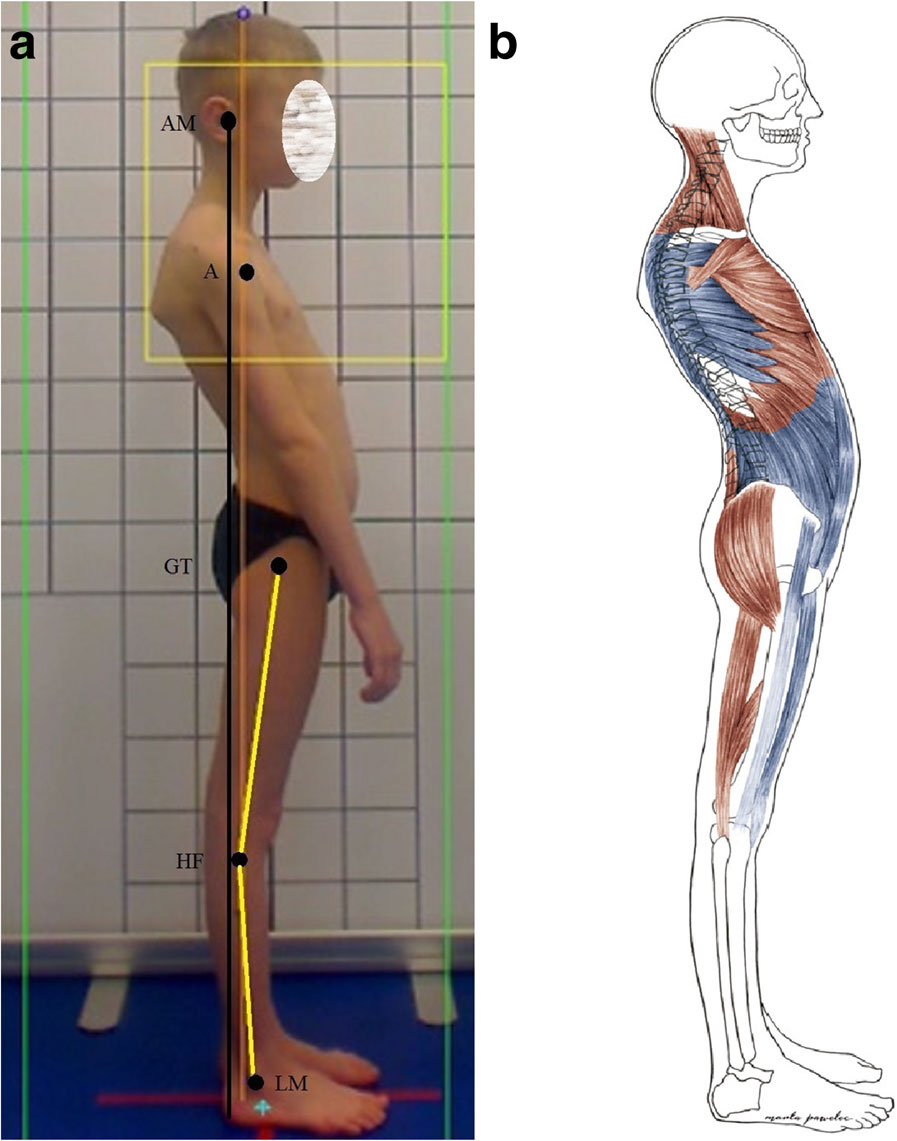
Sway-back posture in a 11-year-old boy. **a** Habitual standing, lateral view. **b** Corresponding schematic representation of the shortened (red) and lengthened (blue) skeletal muscles. Note: AM—external auditory meatus; A—acromion; GT – greater Trochanter; HF—head of fibula; LM—lateral malleolus

The sway-back posture represents a faulty posture that differs from the good one by the following: (1) anterior pelvic shift, (2) thoracic kyphosis extended to the upper part of the lumbar spine (longer thoracic kyphosis is observed), (3) apparently shorter lumbar lordosis, (4) normal or slightly decreased anterior pelvic tilt (Fig. 10) [3, 7, 8, 27].

**Fig. 11:**
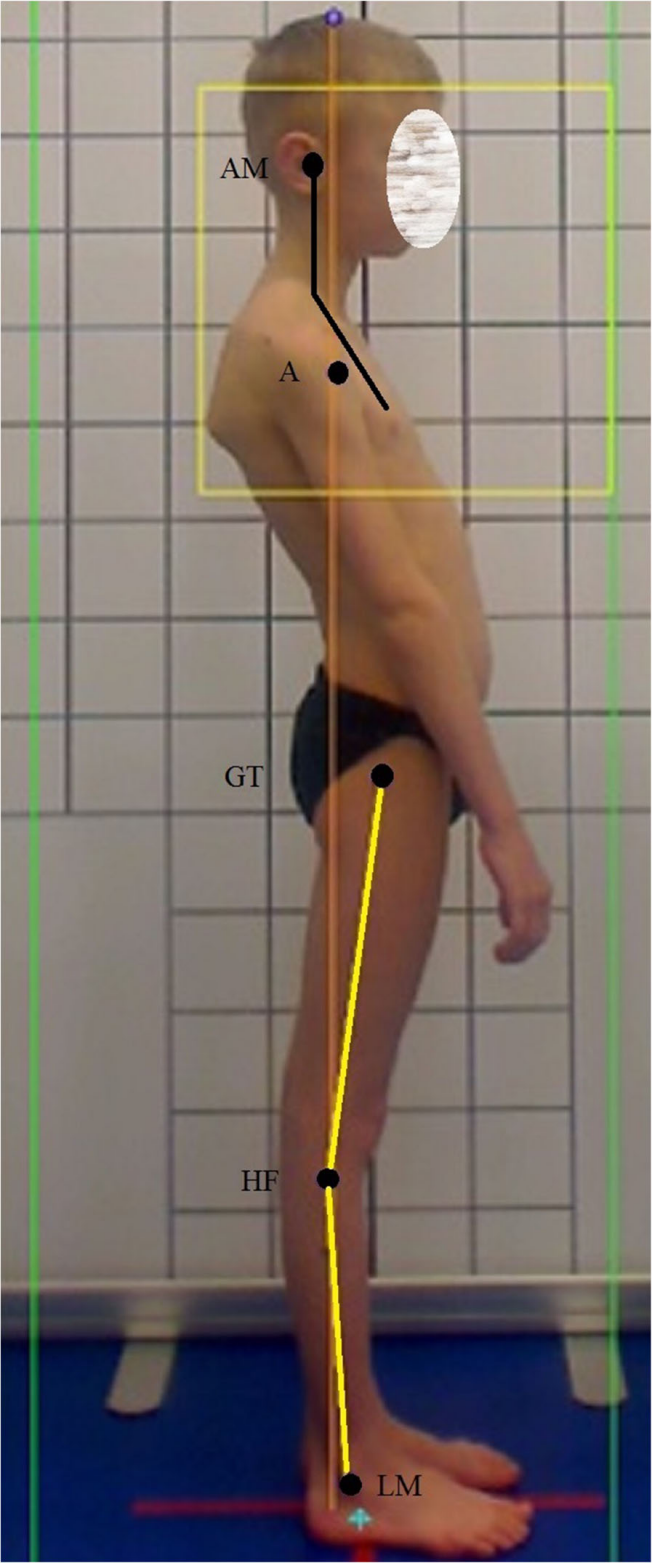
The angle between chest and a head indicates improper head position - protraction. Note: AM—external auditory meatus; A—acromion; GT—greater trochanter; HF—head of fibula; LM—lateral malleolus

In the sway-back posture, the pelvis is in front of the head line, while the upper part of the trunk is usually moved posteriorly to this axis. The head line and the base line usually overlap each other suggesting the normal position of the head. However, the head is in a protraction because of the chest position that is in inclination in relation to the base and the head line [3, 7, 8]. The head line passes posteriorly to the lumbar vertebral bodies (resulting in their extension overload) and posteriorly to the hip joints axis (leading to overload of the hip joints) (Figs. 10 and 11, Table 7) [3, 5].

**Table 7:** The position of body parts in the sway-back posture

Part of the body	Position
Head	Protracted (moved forward)
Cervical spine	Upper part: extended (hyperlordosis) Lower part: flexed (hypolordosis or kyphosis)
Thoracic spine	Upper part: increased flexion (hyperkyphosis) Lower part: normal (kyphosis)
Lumbar spine	Upper part: flexion (kyphosis or hypolordosis) Lower part: increased extension (hyperlordosis)
Pelvis	Shifted anteriorly, decreased anterior tilt
Hip joints	Extended due to decreased anterior tilt of pelvis
Knee joints	Neutral or hyperextended
Ankle joints	Neutral or plantar flexed

#### Functional state of muscles in the sway-back posture

Erector spinae in the upper thoracic and in the upper lumbar part, the muscles that stabilize the scapulae (serratus anterior, lower and middle part of trapezius and rhomboid muscles), abdominal muscles (their lower part), and one-joint (iliacus, psoas), and two-joint hip flexors (rectus femoris, tensor fascia latae) are lengthened [3, 7, 9, 10].

The shortened muscles are suboccipital, sternocleidomastoid, scaleni, chest muscles—pectoralis major and minor, erector spinae lumbar part (lower part), upper fibers of abdominal muscles, gluteus maximus, and hamstrings. All these muscles demonstrate hyperactivity (except for the lower part of lumbar erector spinae, posterior part of internal oblique abdominal muscle, and gluteus maximus) (Table 8, Fig. 10) [3, 7, 9, 10].

**Table 8:** Functional characteristics of muscles in the sway-back posture

Muscle	Lengthened	Shortened	Hypoactive	Hyperactive
Trapezius (middle and lower part)	+		+	
Serratus anterior	+		+	
Rhomboideus major and minor	+		+	
Erector spinae part thoracic (upper part)	**+**			**+**
Erector spinae lumbar part (upper part)	+		+	
Rectus abdominis (lower fibers)	+			+
Abdominal internal oblique (anterior part, lower fibers)	+			+
Abdominal internal oblique (posterior part, lower fibers)	+		+	
Abdominal external oblique (lower fibers)	+			+
Iliacus	+		+	
Psoas	+			+
Two-joint hip flexors	+			+
Suboccipital		+		+
Sternocleidomastoid		+		+
Scaleni		+		+
Trapezius (superior part)		+		+
Pectoralis minor and major		+		+
Erector spinae lumbar part (lower part)		+	+	
Rectus abdominis (upper fibers)		+		+
Abdominal internal oblique (anterior part, upper fibers)		+		+
Abdominal internal oblique (posterior part, upper fibers)		+	+	
Abdominal external oblique (upper fibers)		+		+
Gluteus maximus		+	+	
Hamstrings		+		+

Note: the symbol “+” means that the muscle meets a certain criteria

## Discussion

The aim of the paper was to present the most common types of the sagittal, non-structural misalignments of the human body posture: namely the lordotic posture, kyphotic posture, flat-back posture, and sway-back posture. Each of them may influence both the skeletal and muscular systems leading to the functional disturbances, thus increasing the risk of back and peripheral joint pain or injuries [1–3, 5–8, 10, 19, 28, 29]. The paper was completed with the authors own experience in the diagnosis and treatment of body posture misalignments.

### Clinical relevance—considerations for the correction of non-structural misalignments of body posture

In a good body posture, balance should be maintained between the strength and the flexibility of the antagonistic muscles, for instance, between the hip flexors and the extensors or between the muscles of anterior and posterior part of the pectoral girdle [1–3, 6–10, 23].

According to the classification proposed by Kendall et al., muscles can be assessed in respect to their length and strength. Consequently, e.g., in lordotic posture, among others, the abdominal muscles, the gluteus maximus, the posterior part of gluteus medius, and hamstrings are lengthened. On the other hand, the erector spinae in the lumbar spine, quadratus lumborum, and hip flexors are shortened [3]. The authors suggested that the muscles which are excessive in length are usually weak and require strengthening, while these muscles that are too short are usually strong and maintain antagonistic muscles in a lengthened position [3]. According to our experience, this description of muscle function, based on the direct connection between the lengthening or shortening of muscles and their weakness or strength, respectively, is often taken into consideration while planning corrective exercises by many clinicians. A clinically important question remains: does the muscle lengthening mean that they prove weak, and does the muscle shortening correspond to their increased strength like in the classification proposed by Kendall et al.? The answer was attempted to be given with the detailed classification of muscles presented in the paper [9, 10].

### Muscle hyperactivity can be accompanied by muscle lengthening—the clinical example of hamstrings

**Fig. 12:**
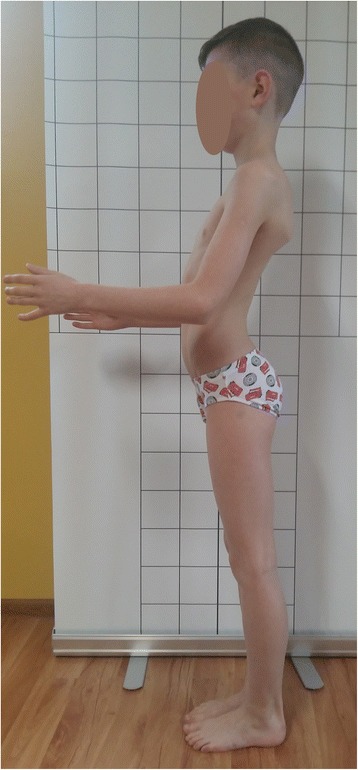
A 10-year-old boy presenting a lordotic posture. Note the following elements: increased lumbar lordosis, increased anterior pelvic tilt

**Fig. 13:**
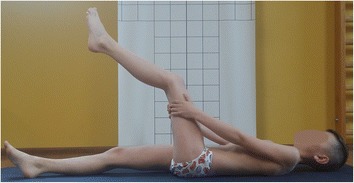
The maximal active knee extension keeping the hip flexed 90°—decreased flexibility of left hamstrings

**Fig. 14:**
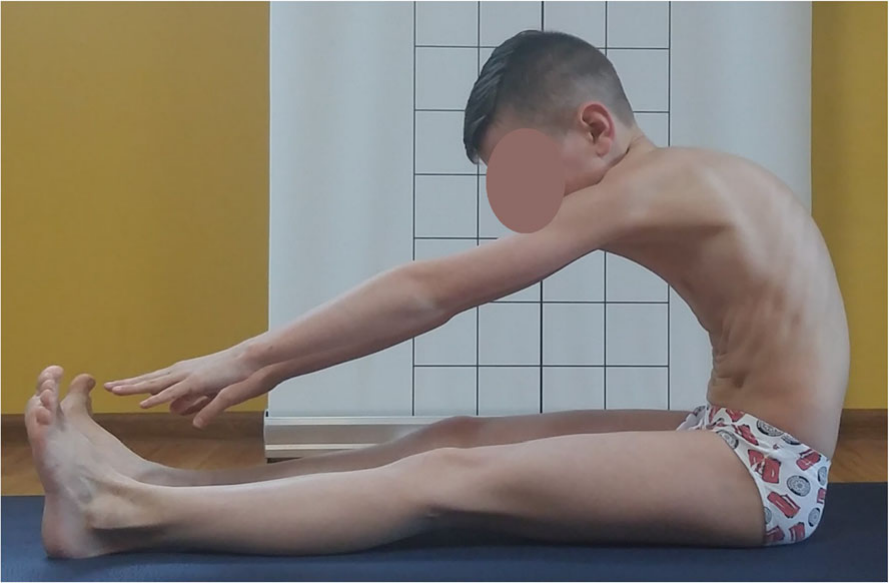
Trunk forward bend test—decreased flexibility of hamstrings and trunk

**Fig. 15:**
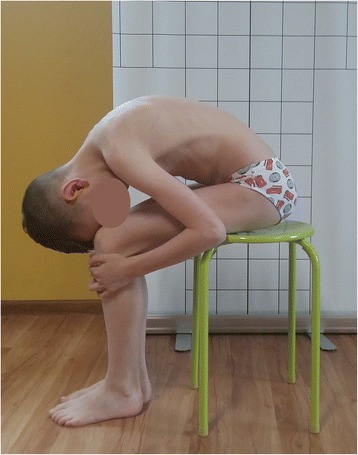
Normal trunk flexion in sitting position with knees flexed

Figure 12 presents a boy whose anterior pelvic tilt is increased, which means that his hamstrings are probably lengthened and, according to Kendall et al., weak. It suggests that during corrective exercises these muscles should be strengthened. However, two functional tests performed by the boy (the long sitting test and the popliteal angle test) reveal interesting information [3, 7, 27]. Figure 13 presents the boy performing the maximal active knee extension while keeping the hip flexed at 90° (the popliteal angle test). The test indicates decreased hamstring flexibility. Accordingly, taking into consideration only the result of this test, the hamstrings would have to be stretched. Figure 14, in turn, shows a maximal trunk forward bending during the long-sitting test. The result of the test suggests the decreasing flexibility of hamstrings and trunk. However, the trunk flexion in sitting on the stool (knees flexed) indicates a good range of motion of the trunk, which confirms the limited flexibility of hamstrings (Fig. 15).

The abovementioned tests revealed the shortening of the hamstrings (Figs. 13 and 14). However, according to Fig. 12 and the description of the lordotic posture in the literature [3, 27], these muscles should be lengthened in this type of faulty posture. This question may be answered with the use of the muscle classification proposed by Bergmark and Richardson et al., which indicates that muscle lengthening may not be related to muscle weakness but can be analyzed in respect to its hyper- or hypoactivity [9, 10]. Taking into consideration the imbalance between the gluteus maximus muscle (hypoactive) and the hamstring muscles (hyperactive) (described detailed in the section “Lordotic posture”), the specific postural physiotherapy should not comprise hamstring exercises aimed at their strengthening (according to Kendall et al. [3]) or stretching (according to functional tests results). The exercises should be focused rather on reducing their activity through regaining the activity of the stabilizers including, in this example, the gluteus maximus [7–10]. Furthermore, the lengthened muscles should not be simply strengthened, but they should be shortened, so the exercises should be performed in the so-called internal (not full) range of motion [10].

In consequence, when planning the corrective exercises, it is important to verify not only the length of the muscles but also their function (hyper- or hypoactivity). It is also important to plan the exercises based on (1) the individual evaluation of the posture, especially when the characteristics of the posture does not match any of the four types presented in the paper, and (2) the analysis of the posture in the usual position for a particular subject in which she/he spends most of the time during a day (e.g., sitting, position at work or while learning, position during learning or hobby).

### Limitations

The authors of this paper focused on the sagittal misalignments of the body posture and their relations with the muscular system. The paper is not discussing the relation of body posture with other factors: psychosocial, nutritional status, structural disorders, or fascial system. We did not discuss the important role of postural education to obtain the good results of corrective exercises.

Further studies are needed to verify a long-term influence of various types of non-structural sagittal misalignments of body posture on the disturbances in the muscular and skeletal system and the functional and structural status of the body.

## Conclusions

1. There exist four principal types of non-structural body posture misalignments in the sagittal plane: lordotic posture, kyphotic posture, flat-back posture, and sway-back posture. Each of them disturbs the physiological loading of the musculoskeletal system, which may lead to functional disorders.
2. In individuals with sagittal misalignments of body posture, not only the evaluation of muscle length or strength should be performed, but also their primary function related to stabilization or mobilization in maintaining good body posture should be taken into consideration.
3. The correction of postural misalignments aimed at the restoration of a good sagittal alignment should start with detailed clinical examination followed by the application of specific corrective exercises directed to recover primary muscles’ function.
